# Research on the Impact Loading and Energy Dissipation of Concrete after Elevated Temperature under Different Heating Gradients and Cooling Methods

**DOI:** 10.3390/ma11091651

**Published:** 2018-09-07

**Authors:** Yue Zhai, Yubai Li, Yan Li, Wenqi Jiang, Xuyang Liu

**Affiliations:** 1 School of Geology Engineering and Geomatics, Chang’an University, Xi’an 710054, China; zy@chd.edu.cn (Y.Z.); liyanlwbdlp@126.com (Y.L.); maomao2163606@126.com (W.J.); xuyangliu@chd.edu.cn (X.L.); 2 School of Earth Sciences and Resources, China University of Geosciences, Beijing 100083, China

**Keywords:** concrete, elevated temperature, impact compression, fractal characteristics, energy dissipation

## Abstract

To provide theoretical basis for fire rescue, post-disaster safety evaluation, and reinforcement of concrete structures, C35 concrete materials are treated with high-temperature heating (200 °C, 400 °C, 600 °C, 800 °C) under two different heating gradients. After natural cooling and water cooling to normal temperature, an impact compression test was carried out at different loading rates using a Split Hopkinson Pressure Bar (SHPB) system with a diameter of 100 mm, and finally the crushed specimens were subjected to a sieving test. The effects of elevated temperatures, cooling methods, heating gradients, and loading rates on the fragment size distribution, fractal characteristics, and energy dissipation of impact-compressed concrete specimens were studied. The results show that with the increase of the loading rate and the rise of the heating temperature, the crushing degree of concrete specimens gradually increases, the average fragment size decreases, and the mass distribution of the fragments move from the coarse end to the fine end. The fragment size distribution of the specimen has obvious fractal characteristics. In addition, its fractal dimension increases with the increase of loading rate and heating temperature, the average size of the specimen fragments decreases correspondingly, and the fracture of the specimen becomes more serious. When the different heating gradients were compared, it was found that the fractal dimension of the specimens subjected to rapid heating treatment was larger than that of the slow heating treatment specimens, and the crushing degree of the specimens with different cooling methods was discrete. By analyzing the energy dissipation of the specimen under different conditions, it is shown that both the fractal dimension and the peak stress increase with the increase of the fragmentation energy dissipation density. It shows that there is a close correlation between the change of fractal dimension and its macroscopic dynamic mechanical properties.

## 1. Introduction

Civil engineering experiences fire accidents during construction and operation. Due to different combustion materials, different structural spaces, and different ventilation conditions, the fire temperature rises at different speeds. At the same time, water-jet fire extinguishing during fire rescue will lead to different degrees of deterioration on the mechanical characteristics of concrete. Therefore, it is of great practical and theoretical value to study the effects of heating rate and cooling method on the impact compression crushing characteristics and mechanical properties of concrete materials after elevated temperature.

At present, fractal theory is commonly used in the theoretical study of the fracture law of rock materials. This theory was founded in the 1970s by Mandelbrot [1]. The research object is the disordered (irregular) and self-similarity system widely existing in nature. Using fractal theory, we can explore some inherent regularity behind hidden phenomena. The interior of the concrete is randomly distributed with many mesoscopic damage structures such as pores, cracks, and weak media. The distribution state and geometry have obvious statistical self-similarity within a certain range of measurement. In addition, under the external load, these mesoscopic damages are rapidly inoculated and merged, so the fragmentation distribution of concrete has certain fractal characteristics. Research in this area has achieved certain results [2,3,4,5,6]. By analyzing the fractal dimension and peak stress of the specimen in the impact compression test, it has a great correlation with the fragmentation energy dissipation density [7,8,9,10]. Xie et al. [11] discussed the intrinsic relationship between energy dissipation, energy release, rock strength and overall damage during rock deformation and failure. It is pointed out that rock deformation and failure is a comprehensive result of energy dissipation and energy release. Carpinteri and Pugno [12] theoretically and experimentally studied the effect of particle size on the energy density and intensity of brittle materials dissipated during compression, and derived the dissipated energy density of structural components under compression.Hu et al. [13] applied the energy dissipation law and brittle dynamic fracture criterion in rock under impact load, and applied the fatigue damage iterative relationship under stress wave to the post-destruction stage of rock, and obtained the relationship between impact energy, rock damage, and block distribution.

Analysis of the existing research results shows that most are static properties of rock materials under room temperature, but the research on the impact fracture morphology, fragmentation distribution, energy dissipation law of elevated temperature-treated materials is relatively lacking. In this paper, the Split Hopkinson Pressure Bar (SHPB) system with a diameter of 100 mm was used to test the impact compression of concrete specimens with different heating gradients and different cooling methods. The crushing specimens were sieved, and fractal theory was used to study the fragmentation distribution law, fractal characteristics, energy dissipation, and its correlation with the corresponding dynamic mechanical properties.

## 2. Materials and Methods 

### 2.1. Specimens Preparation and the Heating and Cooling Test

The material used in the test is C35 concrete provided by a local commodity concrete mixing plant (Xinyida Construction Products Co., Ltd., Xi’an, China). Its main ingredients are Portland cement #42.5, pebbles with diameter of 5 to 25 mm, medium sand (containing about 10% of the mud), fly ash, mineral powder (steel slag powder) and additive (water reducing admixture). The specific mixing ratios are shown in Table 1. The well-mixed concrete materials were poured into the molds and maintained for 28 days in the standard curing room at the room temperature of 18 to 22 °C and with humidity over 95%. The specimens were made into cylinders with diameter of 98 mm and height of 50 mm. Those cylinders were accurately machined by the double-face grinding machine to ensure parallelism error less than 0.2 mm, and surface non-perpendicularity less than 0.2 mm. This experiment has four temperature grades and three loading rates corresponding to two cooling methods and two heating gradients. The test was repeated three times under the same conditions. Thus, the total testing specimens are 144.

**Figure 1:**
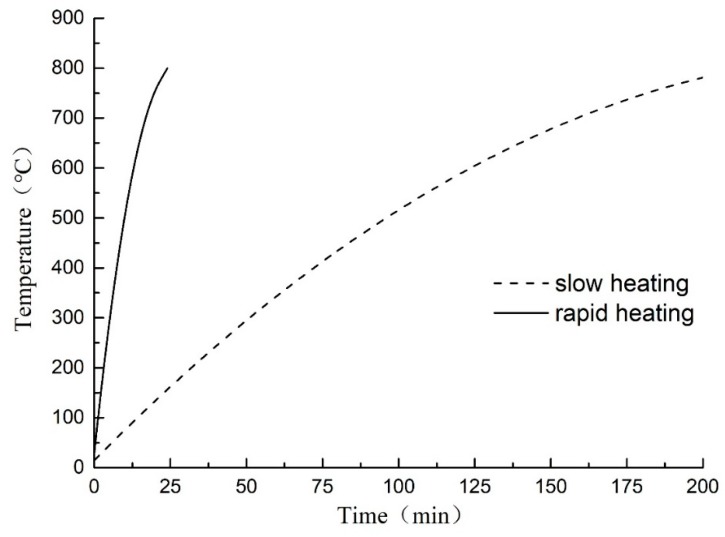
Heating gradient curves.

SX2-10-13 box-type resistance furnace (Sigma Furnace Industry Co., Ltd., Luoyang, China) was used for heating. The working dimensions of the furnace were 400 × 200 × 160 mm^3^, the rated voltage was 380 V, the power was 10 KW, and the highest heating temperature was 1300 °C. Before heating the specimens, the temperature was raised to 100 °C by the thermostat and maintained for 10 min for preheating. Then, the specimens were heated to the set temperatures according to two heating curves as shown in Figure 1. To ensure consistent temperature inside and outside the specimens, they were kept at the set temperature for 2 hours. According to Code for design of sprinkler systems (GB 51251-2017) [14], we assume that 32 °C/min is a rapid fire, that is, a fire caused by flammable and explosive chemicals. 4 °C/min is a slow fire, i.e., a fire caused by the burning of wood products. This test is based on simulating the high-temperature damage of the concrete structure when the fire occurs, and according to the limitation of the furnace equipment, it is assumed that the two heating rates are fast and slow, namely 32 °C/min and 4 °C/min.

**Figure 2:**
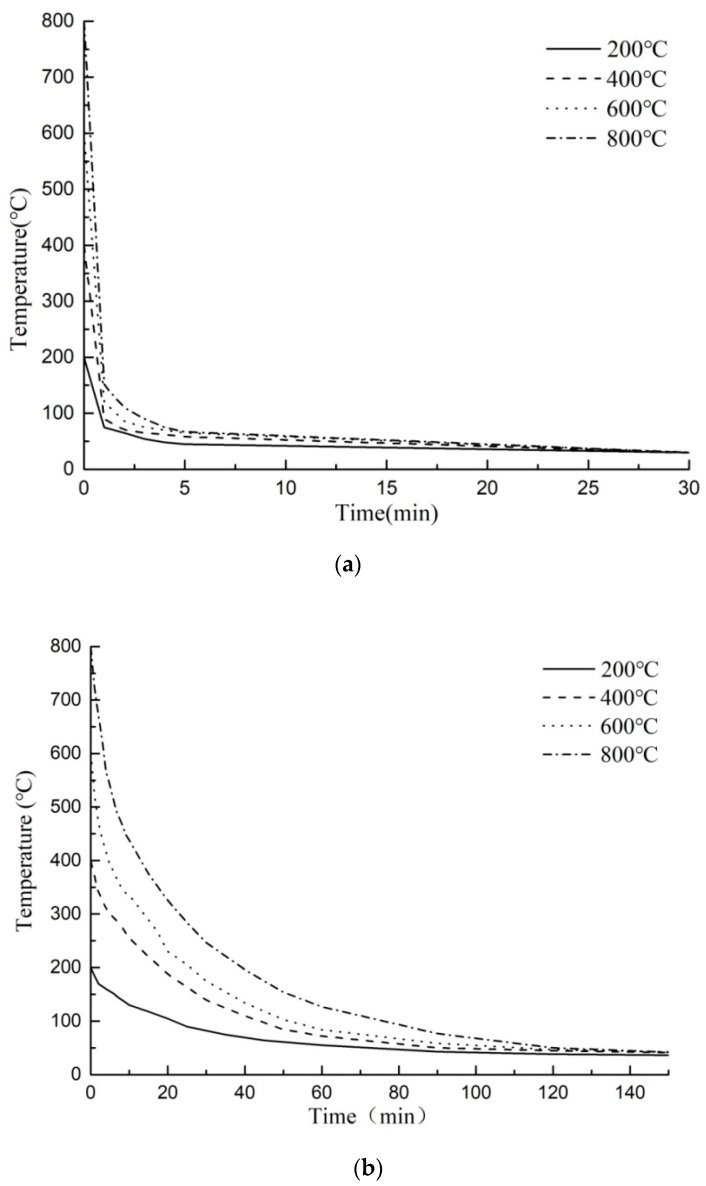
Surface temperature reduction curves of the specimens. (**a**) Temperature reduction curves under water cooling; (**b**) Temperature reduction curves under natural cooling.

The specimens with the natural cooling method were placed in the room temperature environment for natural cooling after heating. Those with water cooling were removed from the heating furnace directly into the cooling water pond at room temperature. The temperature of the pond was measured in real time. When the water temperature remained unchanged, the specimens were taken out and then placed in the room to stand for more than two weeks for natural air dry. During natural cooling, the temperatures of different heating specimens tended to be stable after 60 to 80 min. The temperatures of those through water cooling reduced fast and tended to be stable after 3 to 6 min. The cooling rate of the latter was 20 times faster than that of the former. The temperature reduction curves of the specimens under those two cooling methods were shown in Figure 2. 

### 2.2. Sieving Test on the Specimens after Impact Compression Test

**Figure 3:**
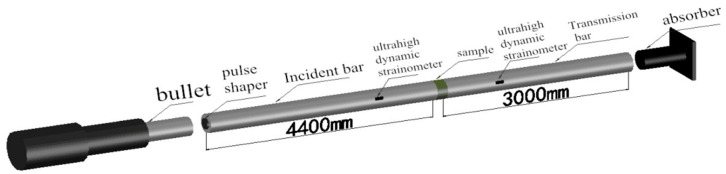
SHPB test system structure.

For the specimens treated with different heating temperatures and cooling methods, the SHPB test system (Liwei Science and Technology Co., Ltd., Luoyang, China) (Figure 3) with a diameter of 100 mm modified by a waveform shaper was used for uniaxial impact compression test. To consider the material strength limit of the equipment and the damage degree of the equipment with high loading rate, the control pressures are set to 0.2 MPa, 0.35 MPa, and 0.5 MPa, that the initial impact rates are 8.56 m/s, 14.23 m/s, and 18.36 m/s.

**Figure 4:**
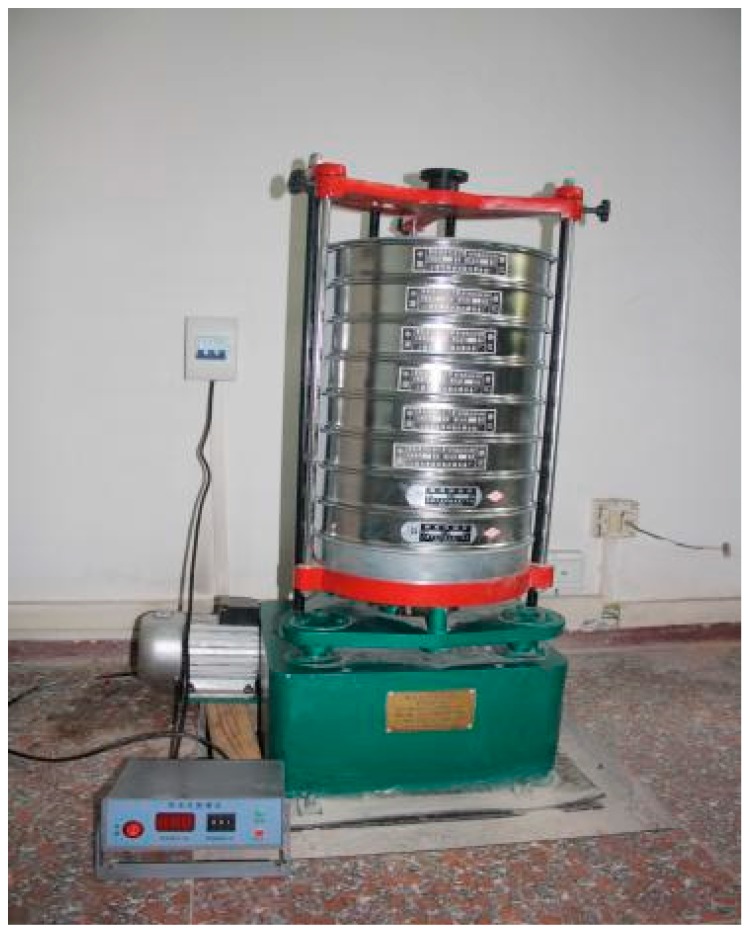
Standard Vibration Pendulum Instrument.

The sieving test was conducted by using the ZBSX-92A shock-type standard vibration pendulum instrument (Xingye Test Instrument Co., Ltd., Cangzhou, China) (shown in Figure 4). The standard square-opening pebble sieve was used as the sieving container with the sieve meshes of 25 mm, 20 mm, 16 mm, 10 mm, 5 mm, 2.5 mm, 1 mm, and 0.5 mm, respectively. The shaking frequency of the equipment was 221 min^−1^; the swing range was controlled to be 25 mm; the vibration frequency was 147 min^−1^; the motor power rated at 0.37 KW. The rotation speed of the three-phase asynchronous motor was 1400 r/min.

According to the regulations in the Coarse Aggregate and Aggregated Mixture Sieving Test Standard (T0302-2005) [15], the aggregates passed through the electric sieve shakers which were arranged according to the mesh size. The particles which had passed over the sieve and the specimens in the next sieve were put together for sieving. The sieves were added manually when necessary until the specimens passed over all sieves. All fine particles adhered to the sieve were removed by using the brush and weighed. After confirming that the mass passing through the meshes within 1 min was less than 0.1% of that of sieve residue, the remaining particles on each sieve were weighed. The sum of the mass of each sieve residue and the mass of sieve residue at the sieve bottom was compared with the total dry mass *m*_0_ of the specimens before sieving. The error should be less than 0.5% of *m*_0_.

## 3. Results and Discussion

### 3.1. Test Phenomenon Analysis

**Figure 5:**
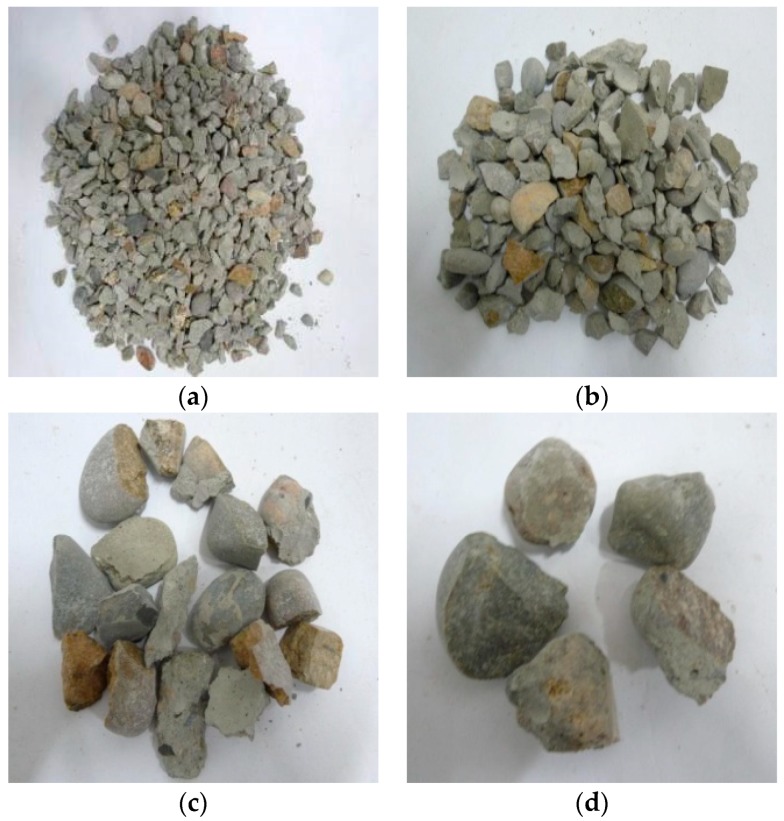
Concrete particles after slow heating up to 200 °C and sieving (impact speed 8.6 m/s), water cooling. (**a**) 5 mm (41.97%); (**b**) 10 mm (37.07%); (**c**) 16 mm (8.93%); (**d**) 20 mm (11.89%).

**Figure 6:**
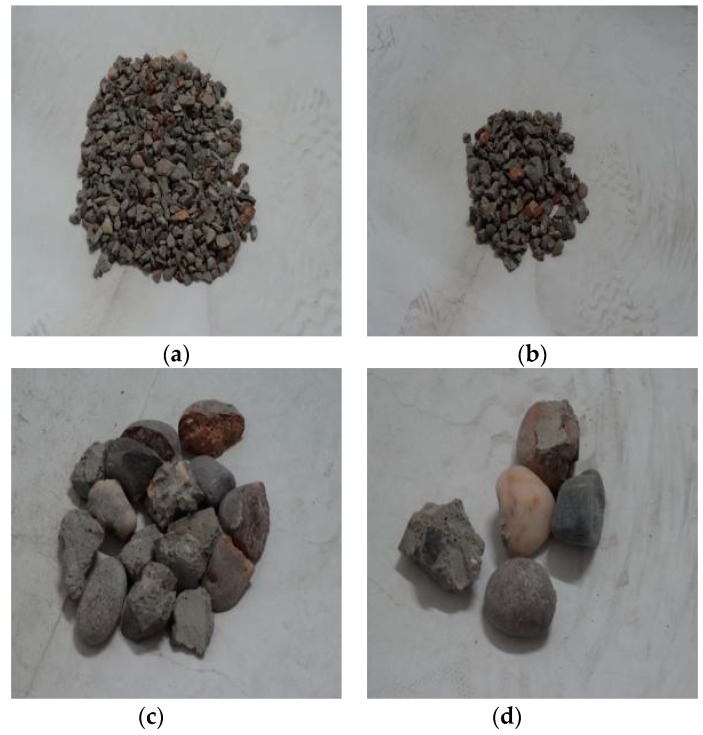
Concrete particles after slow heating up to 400 °C and sieving (impact speed 8.6 m/s), water cooling. (**a**) 5 mm (39.2%); (**b**) 10 mm (33.47%); (**c**) 16 mm (9.07%); (**d**) 20 mm (18.15%).

**Figure 7:**
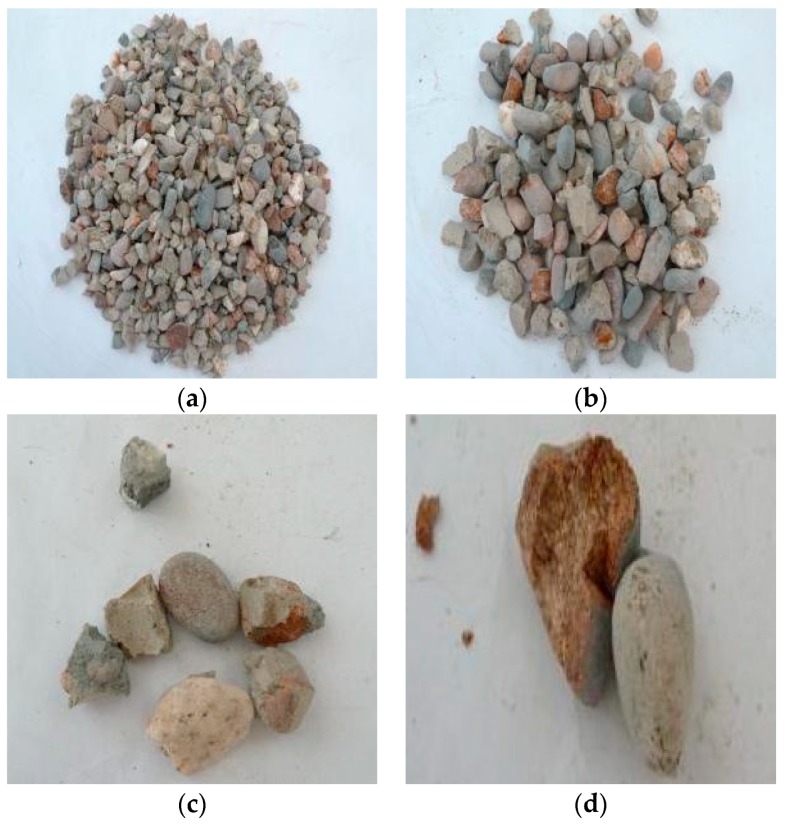
Concrete particles after slow heating up to 600 °C and sieving (impact speed 8.6 m/s), water cooling. (**a**) 5 mm (47.02%); (**b**) 10 mm (23.93%); (**c**) 16 mm (7.44%); (**d**) 20 mm (21.54%).

**Figure 8:**
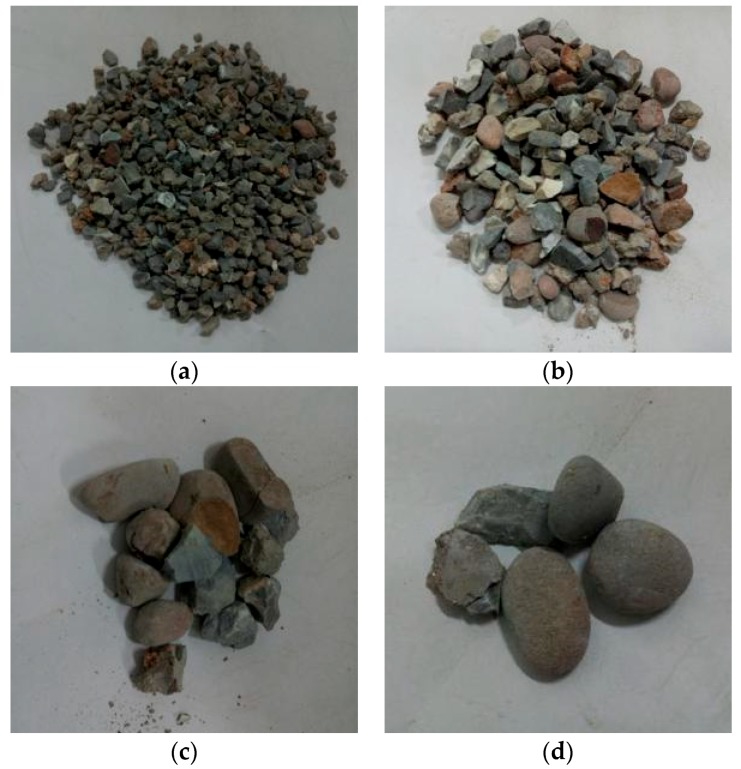
Concrete particles after slow heating up to 800 °C and sieving (impact speed 8.6 m/s), water cooling. (**a**) 5 mm (51.97%); (**b**) 10 mm (26.44%); (**c**) 16 mm (8.2%); (**d**) 20 mm (13.29%).

Through observation of the crushing conditions of specimens under different heating temperatures with the same loading rate and cooling method, the fragments of the concrete specimens at room temperature (25 °C) and being heated at 200 °C were mainly the cement mortar colloid, mostly big particles with complete large coarse aggregate. When the heating temperature rose to 400 °C, the fragments of the specimens were mainly the cement mortar colloid and adhesive interface crushing between coarse aggregate and colloid with many big particles. When the temperature rose to 600 °C, the cement colloid crushed seriously. The crushed particles were small, and a part of coarse aggregate broke into pieces. When the temperature rose to 800 °C, the specimens crushed more seriously. The concrete colloid broke into small particles. The large coarse aggregate crushed seriously and was completely stripped from the cement colloid. Thus, the higher the heating temperature was, the smaller particles (less than 5 mm) the concrete crushed. It indicated that high temperature (above 400 °C) had obvious damage on concrete. Figure 5, Figure 6, Figure 7 and Figure 8 presented the particles after sieving the crushed specimens of different temperatures at the loading rate of 8.6 m/s. The percentages in the figures represent the ratio of the mass of the different sieve pore sizes to the total mass on the sieve.

**Figure 9:**
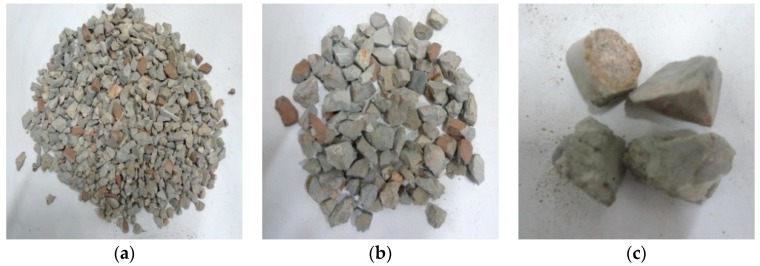
Concrete particles after slow heating up to 600 °C and sieving (impact speed 14.2 m/s), water cooling. (**a**) 5 mm (62.18%); (**b**) 10 mm (23.14%); (**c**) 16 mm (14.62%).

**Figure 10:**
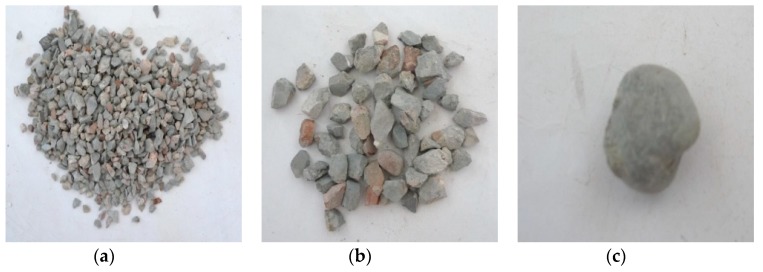
Concrete particles after slow heating up to 600 °C and sieving (impact speed 18.36 m/s), water cooling. (**a**) 5 mm (62.78%); (**b**) 10 mm (23.08%); (**c**) 16 mm (14.08%).

Figure 9 and Figure 10 presented the crushed specimens under water cooling. They were heated up to 600 °C slowly at the loading rate of 14.2 m/s and 18.4 m/s, respectively. According to the figures, when the heating temperature was 600 °C and the impact velocity was 14.2 m/s, the joint between the cement colloid and the aggregate crushed, and the cement colloid broke into small particles. When the impact velocity was 18.36 m/s, the crushed specimens had no aggregate with the size over 20 mm. The aggregate was broken into small particles. The diameters of the crushed particles of the cement colloid were mostly less than 5 mm. The crushing degree of the specimens further increased. It indicated that under the same temperature and same cooling method, the crushing degree of the specimen continued to increase with the loading rate.

**Figure 11:**
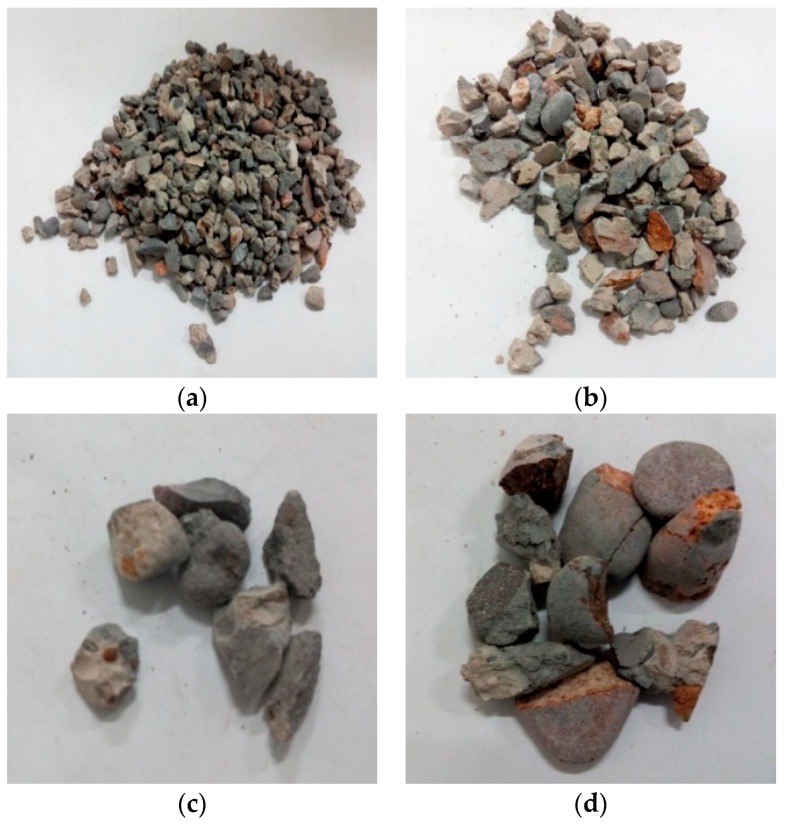
Concrete particles after rapid heating up to 600 °C and sieving (impact speed 8.6 m/s), water cooling. (**a**) 5 mm (53.26%); (**b**) 10 mm (33.59%); (**c**) 16 mm (5.26%); (**d**) 20 mm (7.71%).

Figure 11 presented the crushed specimens with fast heating up to 600 °C and loading rate of 8.6 m/s under water cooling. Compared with those with slow heating in Figure 7, the specimen fragments with rapid heating had more fine particles, and the coarse aggregate showed more obvious cracks under the same temperature, loading rate and cooling method. It indicated that the specimens had serious initial damage.

Thus, the crushing condition of the specimens was affected by not only the heating temperature and loading rate but also the heating rate and cooling method, but the influence of the latter was relatively small. 

### 3.2. Crushing Degree Analysis

Concrete materials showed certain self-similarity in both the random distribution characteristics of the components and the distribution of initial damage. The fractal dimension of their fracture shapes was the function of Weibull uniformity coefficient of the shape coefficient [16]. To make further analysis, the fragmentation distribution function model was studied in the blasting engineering. A series of irregular spheres with different diameters were set. The sieve with the pore size of *r* was used to screen those spheres. The sphere fragments followed certain weight-frequency distribution [17]. The impact fragmentation distribution equation of the rock materials could be obtained from Literature [18,19,20]:

(1) $$Y=M_{r}/M_{T}=(r/r_{m}{)}^{3-D}\;$$

where *r* was the particle size, *r_m_* was the maximum size of the rock, *D* was the fractal dimension of the fragmentation distribution, *M_r_* was the cumulative mass of the fragments with the size less than *r*, *M_T_* was the total mass of the fragments. Natural logarithms were taken on both sides to get the following function:

(2) $$lnY=ln(M_{r}/M_{T})=(3-D)ln(r/r_{m})\;$$

It could be known from Equation (2) that in *ln*(*Mr*/*M_T_*)*~lnr* coordinates, the slope of the fitted line *K* = (*3 − D*), that was

(3) D=3−K 

The fractal dimension of the specimens increased with the rise of the temperature and the loading rate (as shown in Table 2). It indicated that the number of small-size crushing particles increased, and the damage was more serious.

**Figure 12:**
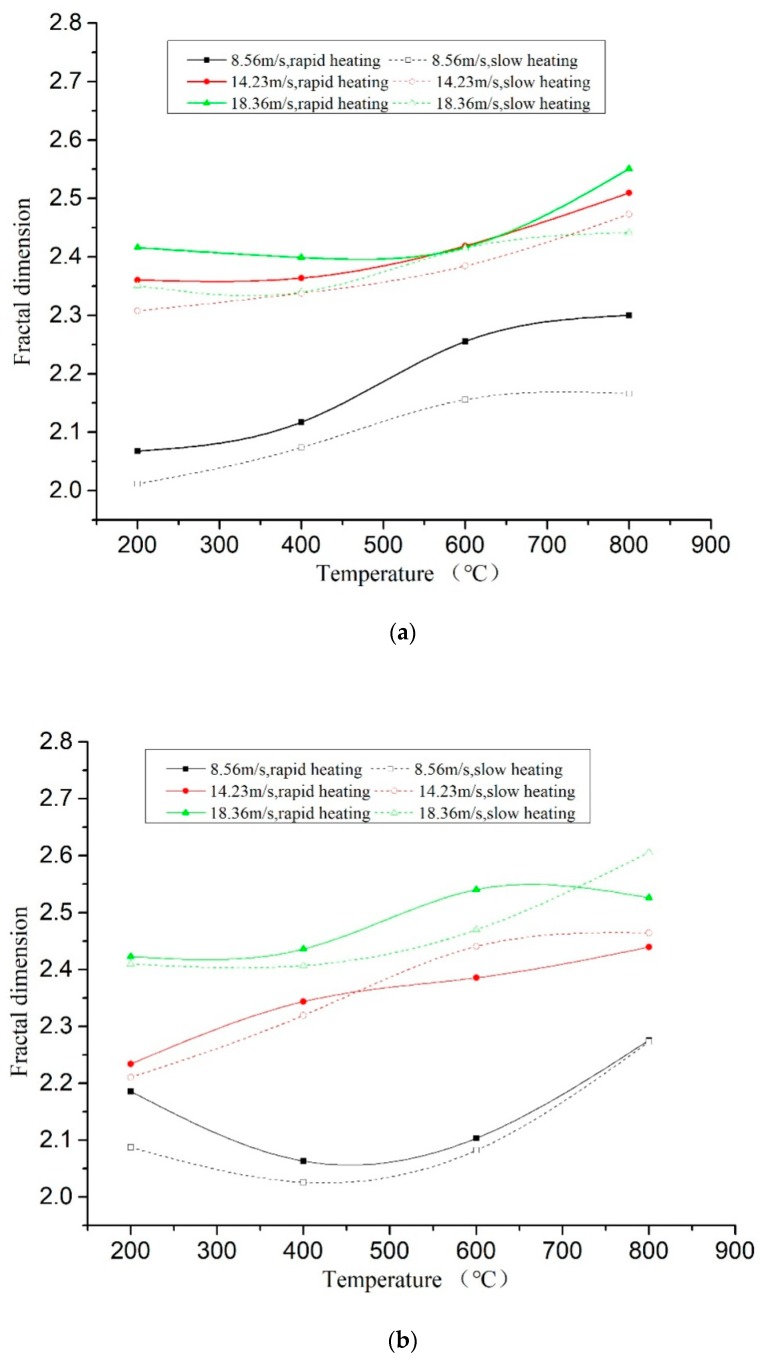
Comparison of the fractal dimensions under different temperature gradients. (**a**) Comparison of specimens under different heating methods, water cooling; (**b**) Comparison of specimens under different heating methods, natural cooling.

**Figure 13:**
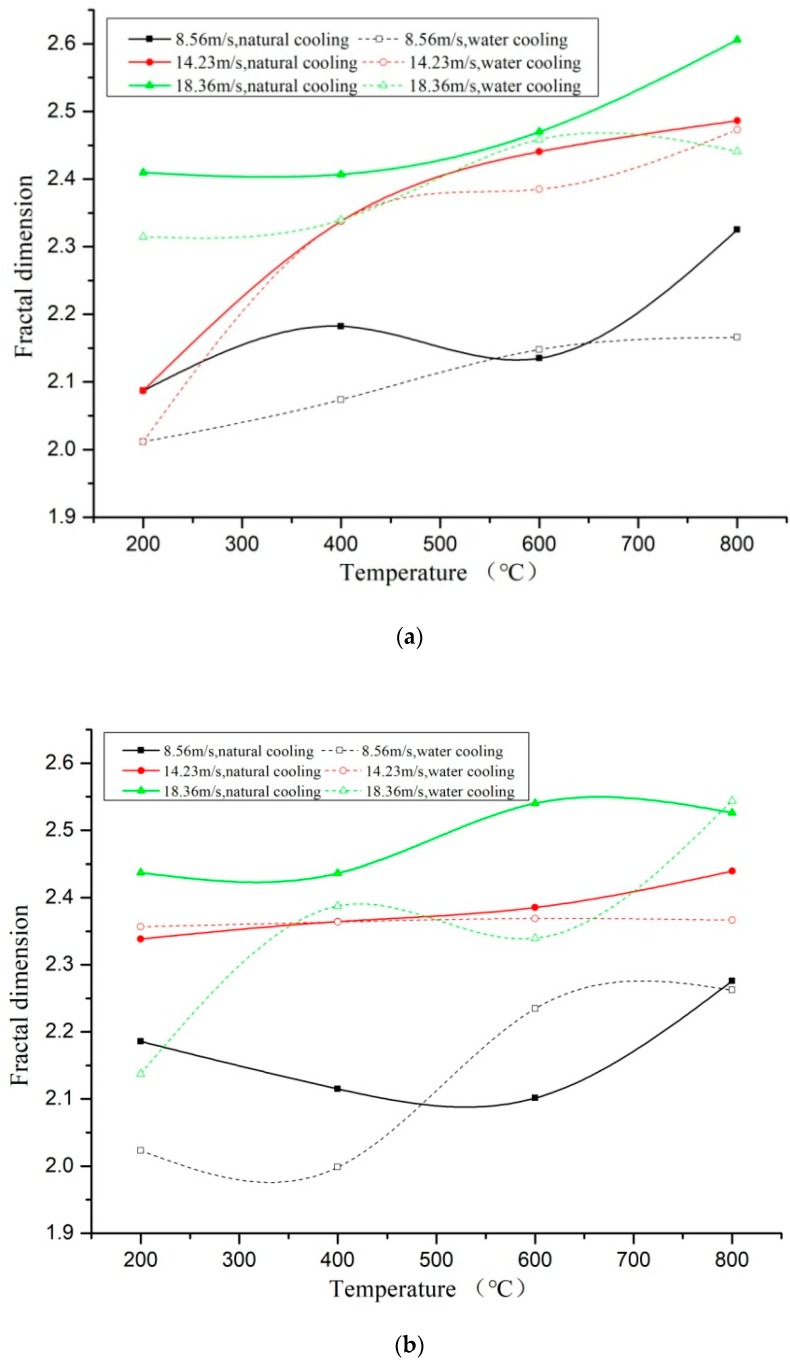
Comparison of the fractal dimensions under different cooling methods. (**a**) Comparison of the fractal dimensions by different cooling methods, slow heating; (**b**) Comparison of the fractal dimensions by different cooling methods, rapid heating.

Figure 12 presented the comparison of the fractal dimensions under two cooling methods and different temperature gradients. It could be found that under the same loading rate and water-cooling method, the fractal dimension of the specimens through fast heating was greater than that of the specimens through slow heating. It indicated that the specimens crushed more seriously when the temperature gradient was big, as shown in Figure 13a. Under natural cooling, the crushing degree of the specimens through fast heating was higher than that through slow heating before 600 °C but was lower than that through slow heating after 600 °C, as shown in Figure 13b. After analysis, when the heating temperature was high (over 600 °C), the damage changed from the physical damage caused by uneven temperature to the chemical decomposition damage caused by the high-temperature heating on the concrete colloid. Slow heating needed a long time, so the temperature damage effect was more obvious, and its influence occupied a main position. 

Under the two kinds of heating gradient conditions, the fractal dimension comparison of the specimens with different cooling methods is shown in Figure 13. It can be seen from the figure that both cooling modes have an upward trend, and the fractal dimension of the naturally cooled specimens is slightly higher than water-cooled, but with some dispersion. Combined with the analysis of the surface temperature reduction curve, during the cooling process, the water-cooling rate is 20 times that of the natural cooling rate, and the temperature of the specimen drops rapidly, causing extreme temperature gradient damage and forming a small range of energy dissipation field around the specimen. The water temperature rises continuously within a certain range, providing a large amount of water for the hydration reaction of the specimen, and partially recovering the original microcrack, so that the concrete integrity is promoted [21]. In general, the fractal dimension shows a certain degree of dispersion under the coupling of cooling method and other multi-factors.

### 3.3. Fragmentation Distribution Analysis

To make further in-depth analysis on the corresponding fragmentation distribution law of the crushed specimens, the retained percentage was calculated on the mass of fragments on each sieve after sieving by Equation (4). The calculation precision was 0.1%.

(4) $$p_{i}=\frac{m_{i}}{m_{0}-m_{s}}\times 100\%\;$$

where *P_i_* is the grader retained percentage on each sieve, *m_s_* is the loss mass caused by sieving, *m*_0_ is the total mass of the dry aggregate for dry sieving, *m_i_* is the grader retained mass on each sieve.

**Figure 14:**
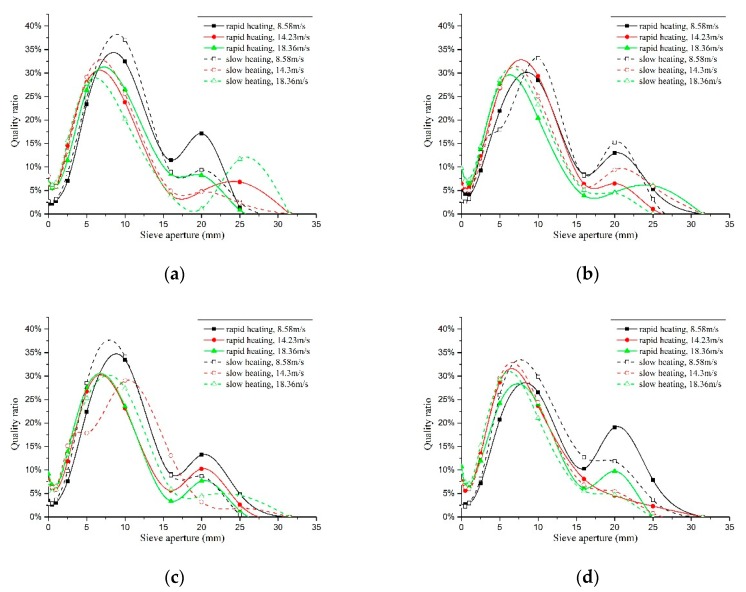

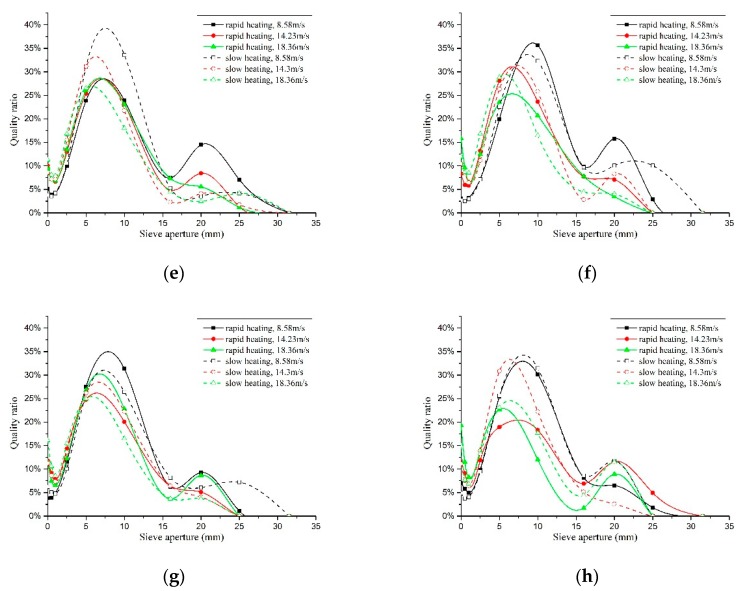
Sieve pore size-mass of the retained on sieve curve under different temperatures. (**a**) 200 °C, water cooling; (**b**) 200 °C, natural cooling; (**c**) 400 °C, water cooling; (**d**) 400 °C, natural cooling; (**e**) 600 °C, water cooling; (**f**) 600 °C, natural cooling; (**g**) 800 °C, water cooling; (**h**) 800 °C, natural cooling.

The accumulative retained percentage of each sieve was the sum of the grader retained percentages of the sieves before it. The passing rates of the sieves were calculated by the residue at the sieve bottom divided by the total mass of the aggregate after deducting the loss, to draw the fragmentation distribution curves under different temperatures and loading rates, as shown in Figure 14. 

At low loading rate (8.6 m/s), the specimen fragments with diameter greater than 10 mm accounted for the largest proportion, between 17% and 38%, and those with diameter within 5 mm accounted for less than 8%. At the high loading rates (14.2 m/s and 18.4 m/s), the specimen fragments with diameter of 5 mm accounted for the largest proportion, between 17% and 32%, and those with diameter less than 5 mm accounted for 10% to 20%. It indicated that the specimens crushed more seriously at the high loading rate.

Under water cooling, the proportion of the specimen fragments with diameter less than 15 mm through fast heating was bigger than that through slow heating, and the proportion of the specimen fragments with diameter greater than 15 mm through fast heating was smaller than that through slow heating up under different loading rates before 600 °C. The situation was just reversed after 600 °C. The fragmentation distribution law under natural cooling was the opposite. It indicated that the crushing laws of the specimens were different under different temperature gradients, which demonstrated the different energy absorption rates of the specimens. Different cooling methods made the energy dissipation of the specimens change rapidly and caused further damage to the concrete specimens, to make the fragmentation distribution law under the impact compression change.

### 3.4. Fragmentation Energy Dissipation Analysis

In the impact compression test, the specimen absorbs the impact energy in a short time, and a part of the energy is transformed into the elastic deformation of the specimen. The rest of the energy causes the initial damage such as microcracks and pores inside the specimen to expand continuously, and new cracks are generated. Therefore, the macroscopic failure of concrete can be considered as the fracture process caused by irreversible absorption and dissipation of energy of internal microcracks when the specimen is subjected to external force [22]. Energy dissipation is not only directly related to the particle size distribution and fractal dimension of the damaged specimen, but also related to the macroscopic mechanical properties. Therefore, the energy dissipation analysis will effectively link the microcrack propagation law with the macroscopic mechanics.

#### 3.4.1. Uniaxial Test and Energy Dissipation Characteristics Analysis

The energy generated in the SHPB test is mainly composed of three parts: incident energy *W_I_*, reflected energy *W_R_*, and transmission energy *W_T_*. The equations are as follow [23,24,25,26].

(5) $$W_{I}=\frac{A_{0}C_{0}}{E}\int \sigma_{I}^{2}dt=A_{0}C_{0}E\int \varepsilon_{I}^{2}dt\;$$

(6) $$W_{R}=\frac{A_{0}C_{0}}{E}\int \sigma_{R}^{2}dt=A_{0}C_{0}E\int \varepsilon_{R}^{2}dt\;$$

(7) $$W_{T}=\frac{A_{0}C_{0}}{E}\int \sigma_{T}^{2}dt=A_{0}C_{0}E\int \varepsilon_{T}^{2}dt\;$$

where $\sigma_{I}$ and $\varepsilon_{I}$ are the stress and strain of the incident wave, $\sigma_{R}$ and $\varepsilon_{R}$ are the stress and strain of the reflect wave, $\sigma_{T}$ and $\varepsilon_{T}$ are the stress and strain of the transmission wave. $A_{0}$ is the cross-section of the bar, E is the elastic modulus of the bar, $C_{0}$ is the propagation wave velocity of the stress wave in the SHPB system, and the equation is:

(8) C0=Eρ 

where ρ is the density of the pressure bar. In this test, $C_{0}$ is 5181 m/s.

Because in the SHPB test, molybdenum disulfide is uniformly applied as a lubricating material on both end faces of the specimen and the contact surface of the SHPB pressure bars. Therefore, the friction between the specimen and the SHPB bar and the energy consumed by the end face friction can be neglected in the calculation. On the other hand, since the concrete specimen is a brittle material, and the volume is much smaller than the pressure bar, the heat energy generated in the impact compression test is relatively small, so it is neglected in the calculation as well. The fragmentation energy dissipation *W* is:

(9) $$W=W_{I}-(W_{R}+W_{T})\;$$

To show the crushing energy dissipation per unit volume of the specimen more clearly, reflecting the characteristics of the fracture of the specimen, judging the crushing energy of the specimen under different conditions, it is considered that the energy dissipation density is used to reflect the energy absorption of the specimen. In addition, the energy dissipation per unit volume of rock absorption and crushing can be calculated by the following equation:

(10) $$\zeta =\frac{W}{V}\;$$

where ζ is fragmentation energy dissipation density, and V is the volume of specimen. 

#### 3.4.2. Energy Dissipation Analysis of Different Temperature Gradients

**Figure 15:**
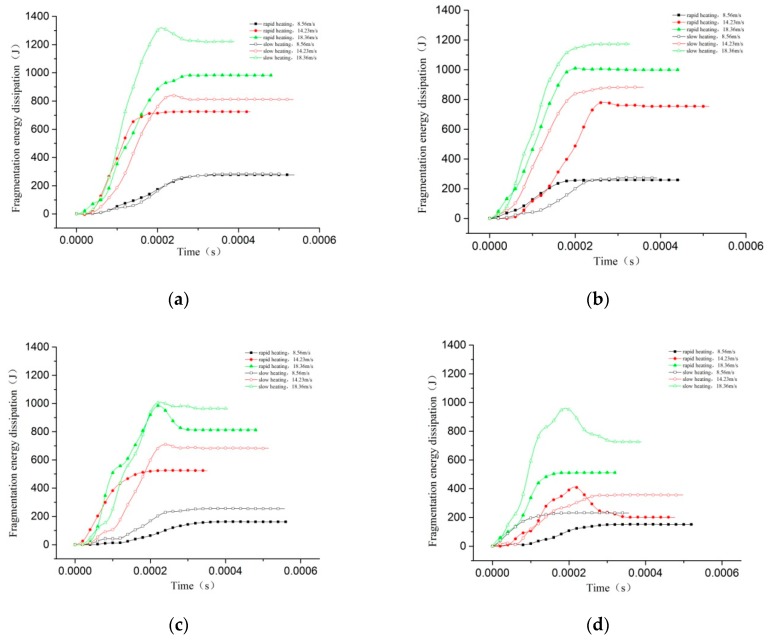
Energy dissipation curve under different temperature gradient conditions. (**a**) 200 °C; (**b**) 400 °C; (**c**) 600 °C; (**d**) 800 °C.

The crushing energy dissipation of the impact test specimens treated under the same temperatures, the same cooling methods, and different heating gradient conditions is compared, as shown in Figure 15.

It can be seen from the figure that under different conditions, the energy dissipation of two heating methods specimens increases with the increase of the impact velocity, but the energy dissipation of the slow heating specimens is greater than that of the rapidly heated specimens. The main reason is that the temperature difference is small between inside and outside of the specimen when the temperature is raised slowly. The initial damage caused by the temperature on the specimen is small. It was found that by increasing the size of the cluster of the specimen, the width of the cracks also increases when the temperature rises rapidly. The temperature gradient damages the specimen more severely, so the energy absorbed during the crushing is relatively small [27].

#### 3.4.3. Energy Dissipation Analysis of Different Cooling Methods

**Figure 16:**
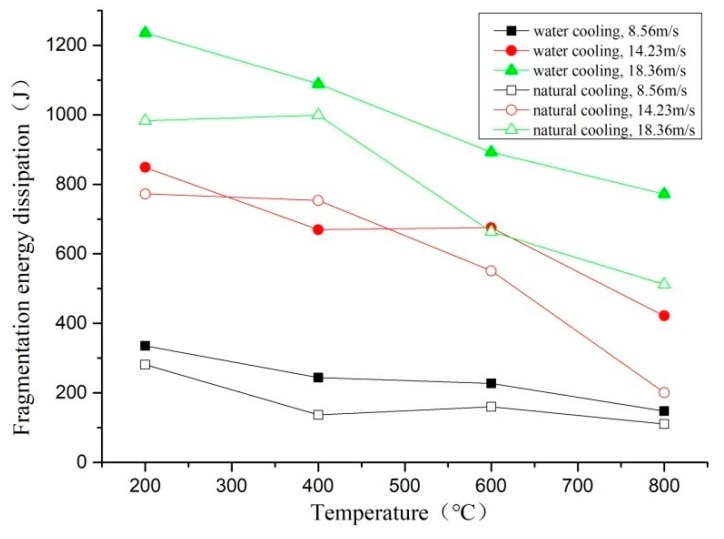
Fragmentation energy dissipation of different cooling methods.

Under different cooling methods, the cooling rate of concrete specimens has a large difference, and the energy dissipation of impact compression is shown in Figure 16.

It can be seen from the above figure that the fragmentation energy dissipation of the two cooling methods decreases with increasing temperature and increases with the increase of the impact velocity. Under the same temperature and the same impact velocity, the energy dissipation of the water-cooled specimen is higher than that of the naturally cooled specimen, but it still has a certain dispersion. This conclusion is consistent with the fractal dimension and fragmentation distribution in the previous analysis. It is indicated that the specimen is cooled in water after heating at high temperature, at this time, the concrete material reacts with water, and the damaged concrete material is repaired to a certain extent [28]. In the SHPB impact compression test, the energy absorption is larger, but the crack development diffusion area is smaller, so the fracture of the specimen requires more energy dissipation. The specimens under natural cooling conditions have serious initial damage. During the cooling process, the specimens are further dehydrated and oxidized, which further increases the damage of the specimens. The specimens absorb energy in the SHPB impact test, so the crushing of the specimen requires less energy dissipation.

### 3.5. Fragmentation Energy Dissipation Density Characteristics and Peak Stress Analysis

**Figure 17:**
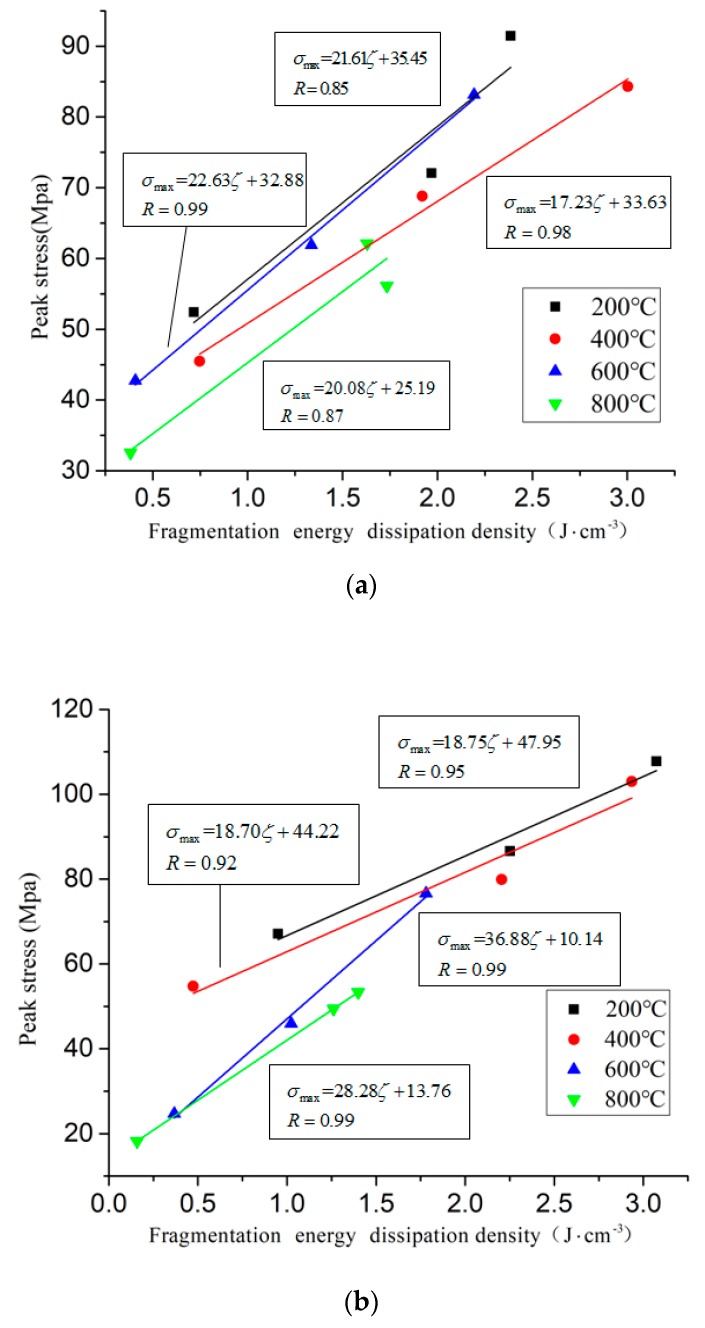
The linear relationship between fragmentation energy dissipation density and peak stress under different conditions. (**a**) Rapid heating and natural cooling (**b**) Slow heating and water cooling.

According to the law of fragmentation energy dissipation density and the uniaxial impact peak stress, the relationship between the fragmentation energy dissipation density and the peak stress is linear fitted, as shown in Figure 17.

It can be seen from the figure that the peak stress has a good linear relationship with the fragmentation energy dissipation density, and the peak stress increases with the increase of the fragmentation energy dissipation density. The fitting line shows an upward trend, and the fragmentation energy dissipation density in the fitted line is positively correlated with the peak stress. In the fitting equation, the slope represents the rate of increase of the peak stress with the energy absorbing, and the fitting slope of the specimen is between 15–37. The peak stress of the specimen is not completely determined by the energy dissipation, but by the multi-factor coupling. Different factors have different strength and weakening effect on the specimen. The increase rate of the peak stress is a multi-factor nonlinear coupling competition mechanism.

### 3.6. Fragmentation Energy Dissipation Density Characteristics and Fractal Dimension Analysis

**Figure 18:**
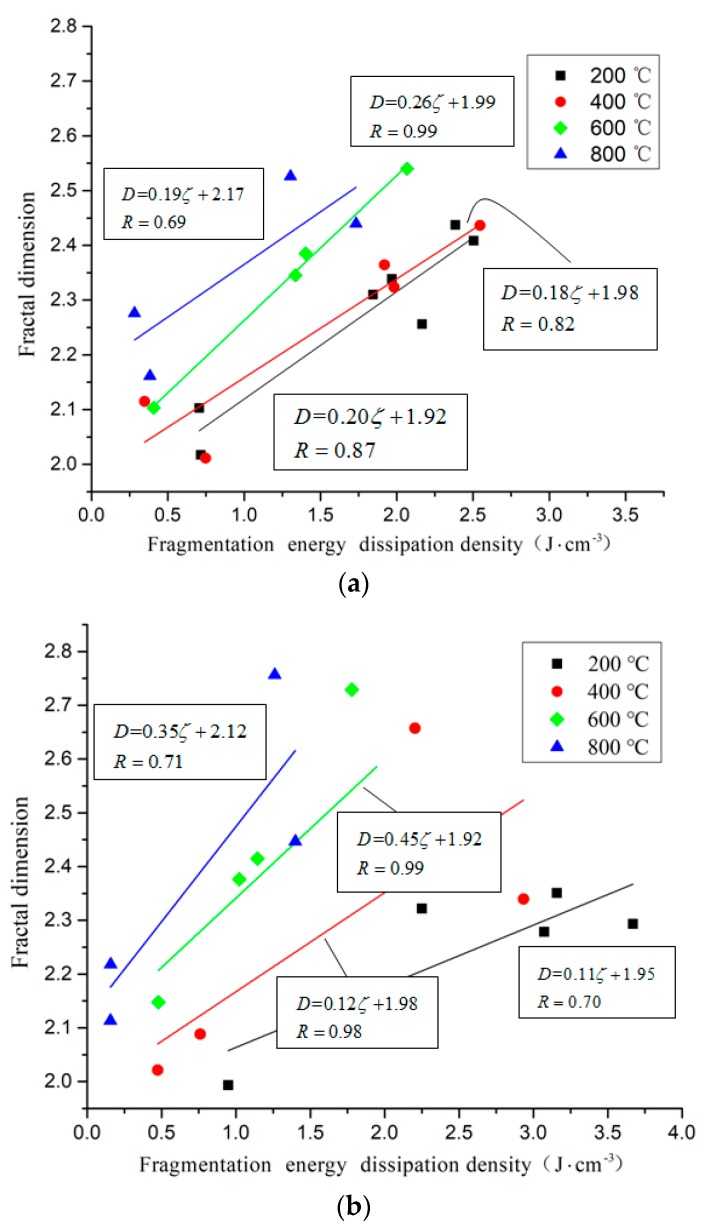
The linear relationship between fragmentation energy dissipation density and fractal dimension under different conditions. (**a**) Rapid heating and natural cooling (**b**) Slow heating and water cooling.

According to the law of the fragmentation energy dissipation density and fractal dimension, the relationship between the fragmentation energy dissipation density of the concrete specimen and the fractal dimension is linear fitted, as shown in Figure 18.

It can be seen from the above figure that under the same cooling method and temperature gradient, the fractal dimension of the fractured specimen increases with the increase of energy dissipation density, and the energy dissipation density is positively correlated with the fractal dimension. According to the difference in temperature, the specimen treated at a higher temperature has a higher fractal dimension when the energy dissipation density is smaller than that of the low temperature-treated specimen. In the fitting equation of the relationship between the energy dissipation density of the fractured specimen and the fractal dimension, the slope represents the increase of the fractal dimension with the energy absorption. The fitting slope of the specimen is between 0.11 and 0.45. When the other conditions are the same, the slope of the fitting equation after 600 °C is significantly increased, because Ca(OH)_2_ in concrete begins to decompose to form CaO at 547 °C, and Ca(OH)_2_ will be formed when water is encountered. In addition, the volume will expand by 44% [29,30]. More and new microcracks are formed in the specimen, while the original cracks are expanded, widened and connected. It is indicated that during the crushing process, the temperature has a major influence on the fracture of the specimen, and the high temperature makes the specimen load capacity greatly weakened.

## 4. Conclusions 

Different loading rate impact compression tests were carried out on C35 concrete specimens treated with different heating gradients and different cooling methods, and the crushed specimens were sieved and analyzed. Through the study of energy dissipation, the relationship between fracture and macroscopic mechanical properties is analyzed and the following conclusions are obtained:

The crushing of the concrete specimens with low heating temperature (less than 200 °C) and low loading rate was mainly the crushing of cement mortar colloid. They were mostly the big particles with complete large coarse aggregate. With the rise of the temperature (over 400 °C), the crushing of the adhesive interface between coarse aggregate and colloid intensified. At the same time, the cooling method and the temperature gradient had an obvious impact on the crushing law. 

With the increase of influencing factors such as strain rate, heating temperature and temperature gradient, the fracture degree of the specimen increases, the number of fragments increases, the size decreases, and the fractal dimension of the fragmentation increases gradually. At the same time, the cooling method has a certain influence on the fracture of the specimen, but the regularity is weak.

By analyzing the energy dissipation of the specimen, it is shown that both the fractal dimension and the peak stress increase with the increase of the fragmentation energy dissipation density, but different heating gradients and cooling methods have a significant influence on the relationship between them, and the final performance is determined by a nonlinear competitive coupling mechanism formed by various intensifying and weakening factors.

## Figures and Tables

**Table 1:** Mixing Proportions of the Concrete Specimens.

Item	Water	Cement	Pebble	Sand	Fly Ash	Mineral Powder	Additive
Ingredients (kg·m^−3^)	155	250	1165	670	90	60	10
Proportion	0.62	1.00	4.66	2.68	0.36	0.24	0.04

**Table 2:** Fractal Dimension of Crushing Concrete.

Loading Rate (m/s)	Fractal Dimension			
	200 °C	400 °C	600 °C	800 °C
8.56	2.0872	2.0261	2.0822	2.3250
14.23	2.2766	2.3376	2.4406	2.4861
18.36	2.4096	2.4068	2.4699	2.6058
